# *TXM-Sandbox*: an open-source software for transmission X-ray microscopy data analysis

**DOI:** 10.1107/S1600577521011978

**Published:** 2022-01-01

**Authors:** Xianghui Xiao, Zhengrui Xu, Feng Lin, Wah-Keat Lee

**Affiliations:** a National Synchrotron Light Source II, Brookhaven National Laboratory, Upton, NY 11973, USA; b Department of Chemistry, Virginia Tech, Blacksburg, VA 24061, USA

**Keywords:** transmission X-ray microscope, data analysis, computer programs, X-ray imaging, XANES

## Abstract

A transmission X-ray microscope (TXM) can investigate morphological and chemical information of a tens to hundred micrometre-thick specimen on a length scale of tens to hundreds of nanometres. It has broad applications in material sciences and battery research. TXM data processing is composed of multiple steps. A workflow software has been developed that integrates all the tools required for general TXM data processing and visualization. The software is written in Python and has a graphic user interface in *Jupyter Notebook*. Users have access to the intermediate analysis results within *Jupyter Notebook* and have options to insert extra data processing steps in addition to those that are integrated in the software. The software seamlessly integrates *ImageJ* as its primary image viewer, providing rich image visualization and processing routines. As a guide for users, several TXM specific data analysis issues and examples are also presented.

## Abstract 2

*TXM-Sandbox*, a workflow software that integrates all the tools required for general transmission X-ray microscope data processing and visualization, is presented. The software is written in Python and has a graphic user interface in *Jupyter Notebook*.

## 1. Introduction

X-ray microscopy techniques provide position-sensitive structural and chemical information of samples with various modalities, *e.g.* element-sensitive fluorescence (Kopittke *et al.*, 2018 ▸; Pushie *et al.*, 2014 ▸), crystalline structure sensitive diffraction (Thibault & Elser, 2010 ▸; Bernier *et al.*, 2020 ▸; Hong *et al.*, 2020 ▸; Xu, Hou *et al.*, 2020 ▸) and chemical-state-sensitive near-edge absorption spectroscopy (Kuzmin & Chaboy, 2014 ▸). Such information at mesoscales is complementary to their counterparts of X-ray bulk techniques at macroscopic scales and electron-microscopy techniques at microscopic scales, which makes X-ray microscopy critical in studying complex systems that have hierarchical structures (Lin *et al.*, 2017 ▸; Bak *et al.*, 2018 ▸).

Amongst various X-ray microscopy techniques, transmission X-ray microscopes (TXM) are capable of providing fast spectro-imaging capabilities at the tens of nanometre scale (Spence *et al.*, 2021 ▸). Moreover, TXM can provide fast measurements of morphological and chemical structural information of samples. Thus, TXM has been utilized to study various energy storage materials and engineering materials under both dynamic and static conditions (Spence *et al.*, 2021 ▸; Bak *et al.*, 2018 ▸; Lin *et al.*, 2017 ▸).

TXM data processing depends on the type of data. Different types of TXM require different analysis processes and prerequisites for each individual process step. It is highly desired to have software that integrates all types of TXM data analyses with guides through each type of analysis. There are a few software programs either dedicated to or capable of TXM data analysis. *TXM-Wizard* is a well known program with a GUI for TXM data analysis based on MATLAB (Liu *et al.*, 2012 ▸). It integrates many useful pre-processing and XANES analysis tools. However, the program is closed, so it is not easy for users to have the freedom to tune the routines according to the properties of the specific input data. It also often requires MATLAB to process intermediate results, which is not convenient for users who do not have MATLAB licenses. *PyXAS* is a Python-based program with a GUI for TXM two-dimensional and three-dimensional XANES (2D XANES and 3D XANES) analysis (Ge & Lee, 2020 ▸). Although it is an open-source program, it is not straightforward for users to integrate their customized routines into it without modifying its GUI layout. *PyXAS* also lacks tomography data processing integration that makes its 3D XANES dependent on other tomography data processing packages. *MANTiS* is another Python-based program with a GUI for spectroscopy data analysis (Lerotic *et al.*, 2014 ▸). For TXM data analysis purposes, it can only handle 2D XANES analysis. Many researchers use different independent customized routines in their workflow to process their data. For instance, researchers use Python scripts based on *PyMCA* and *Silx* packages to process 2D XANES data (Gambardella *et al.*, 2020 ▸). While such a piece-wise approach provides maximum flexibility and use of every available software package, it requires the researcher to tackle the specific interface issues between the various packages and their data. Such an approach is not ideal for a user facility such as NSLS-II where there is a need to provide users with a common user-friendly tool for data analysis.

*TXM-Sandbox* is developed to provide users with a convenient graphic user interface and flexibility, allowing users to plug in the customized operations in the middle of the data analysis flow. It targets overcoming the limitations in the currently available packages. The paper is organized as follows: the software design considerations are described in Section 2, the detailed structure of *TXM-Sandbox* is shown in Section 3, the application examples are presented in Section 4, and a short discussion on the future plans in Section 5 closes the paper.

## 2. Software design consideration

Python is chosen for building *TXM-Sandbox*. Python has a large user community and a growing ecosystem. Numerous Python libraries are available for scientific data analysis purposes that provide *TXM-Sandbox* users great flexibility to develop customized plugins upon the tools built in *TXM-Sandbox*. *Jupyter* with Python as its back-end provides an interactive Python environment (Kluyver *et al.*, 2016 ▸). *ipywidgets* are interactive HTML widgets for *Jupyter* notebooks and the IPython kernel (https://github.com/jupyter-widgets/ipywidgets). It provides rich widget components for building graphic user interfaces (GUIs) inside *Jupyter*. Building the *TXM-Sandbox* GUI with *ipywidgets* curates data objects inside *Jupyter*, making them accessible not only within the *TXM-Sandbox* but more generally within *Jupyter*. Therefore, *TXM-Sandbox* presents users with an interface that allows them to directly access and analyze the intermediate results in the middle of their data analysis workflow. The change to the data directly takes effect in the processing operations inside *TXM-Sandbox*. Besides, *TXM-Sandbox* generates Python script files for the major computation steps in each type of analysis, *e.g.* reconstruction for tomography, and image alignment and spectrum fitting for 2D/3D XANES analysis. Users can insert extra data processing steps in the scripts to test the routines. Such a framework provides users an easy way to develop their own customized data processing plugins that can be integrated into *TXM-Sandbox* in future releases.

As an image-based data analysis software, image visualization is a critical component. *ImageJ/Fiji* is a popular image processing and visualization software widely used in imaging communities that integrate numerous image visualization and processing tools (Schneider *et al.*, 2012 ▸; Schindelin *et al.*, 2012 ▸). While *ImageJ/Fiji* is Java based, a Python wrapper package *pyimagej* (https://github.com/imagej/pyimagej) provides bi­lateral communication between Java and Python that allows *TXM-Sandbox* to use *ImageJ/Fiji* as its primary image viewer and accessory image processor. By building *TXM-Sandbox* based on *Jupyter Notebook* and *ImageJ/Fiji*, it combines the best of both worlds into an ecosystem with great expansibility.

## 3. Software structure

**Figure 1:**
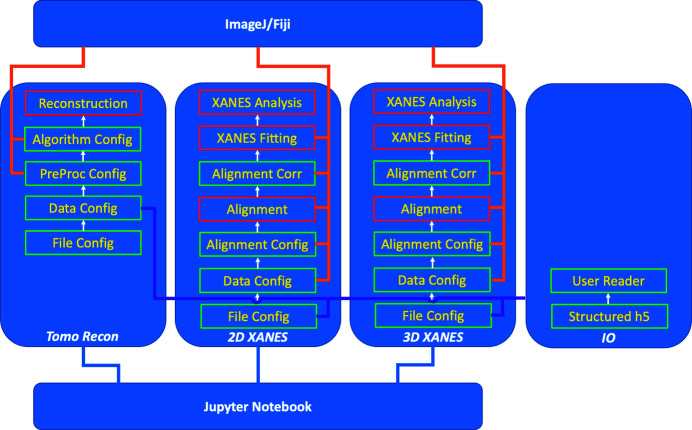
*TXM-Sandbox* software structure. Python scripts are generated in the steps marked with red boxes.

Fig. 1 ▸ illustrates the software structure. *TXM-Sandbox* currently includes three data analysis modules: tomographic reconstruction, 2D XANES, and 3D XANES, and an input/output (I/O) configuration module. Each analysis module is composed of (1) data input/output configuration, (2) data pre-processing, and (3) data-type-specific analysis. In general, a type of data analysis needs to follow a certain procedure since a given operation can only be applied to the data after the required pre-processing. *TXM-Sandbox* has a logic control function for each type of analysis to manage the data dependency relations. The logic function enables/disables each operation according to the current state of the processed data. Therefore, it guarantees users follow the proper order over the analysis process for each type of data. *TXM-Sandbox* generates Python script files at the steps marked in red boxes in Fig. 1 ▸. Users can use these scripts as templates to process multiple tasks in batch or insert user-customized routines for processing results with improved quality.

### 3.1. Customizable I/O module

**Figure 2:**
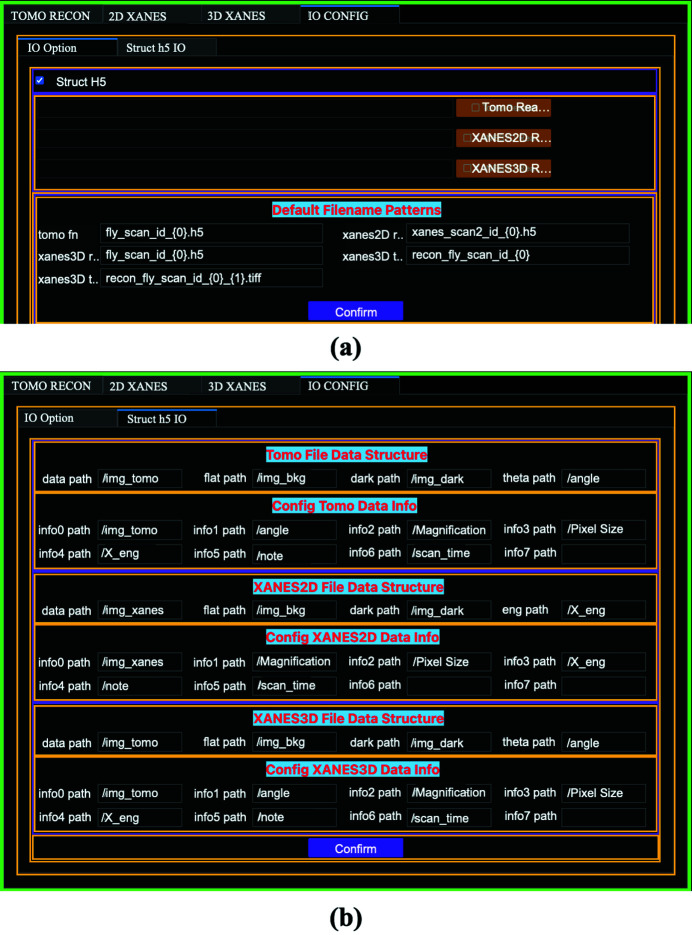
Customizable I/O module configuration. (*a*) Reader configuration; users can define customized readers for each type of data or use the default HDF5 file format. (*b*) HDF5 file structure configuration.

The data from different sources may be saved in different file formats with different file structures. An I/O module is designed to accommodate these variations. Fig. 2 ▸ shows the *TXM-Sandbox* layout. The I/O module is under its own tab (‘IO Config’). The default data file format is Hierarchical Data Format, version 5 format (HDF5), which is a data model, library, and file format for storing and managing data (http://www.hdfgroup.org/HDF5) and widely used in the X-ray user community. Under the ‘IO Config’ tab, users can define the input and output file name templates [Fig. 2 ▸(*a*)] and internal paths to the datasets for each type of data [Fig. 2 ▸(*b*)]. In the case of tomography, the key datasets are dark-field, flat-field, the sample’s projection images, and the associated angle positions. Fig. 2 ▸(*b*) shows the paths to these four key datasets defined in the files acquired at National Synchrotron Light Source II at Brookhaven National Laboratory, USA. These paths might be defined differently at different synchrotron facilities. Users can configure these paths for the data acquired at different lightsources. Users can also specify what metadata to be extracted and define the internal paths to the selected metadata [Fig. 2 ▸(*b*)]. In the instance of tomography, users may like to display the magnification of TXM, pixel size, X-ray energy, projection data size, rotation angle range, date and time of the measurement, and user’s experimental notes. For file formats other than HDF5, customized readers with standard input/output interfaces can be implemented per users’ requirements. Currently there are no customized readers implemented in *TXM-Sandbox* yet.

### 3.2. Tomography GUI layout

**Figure 3:**
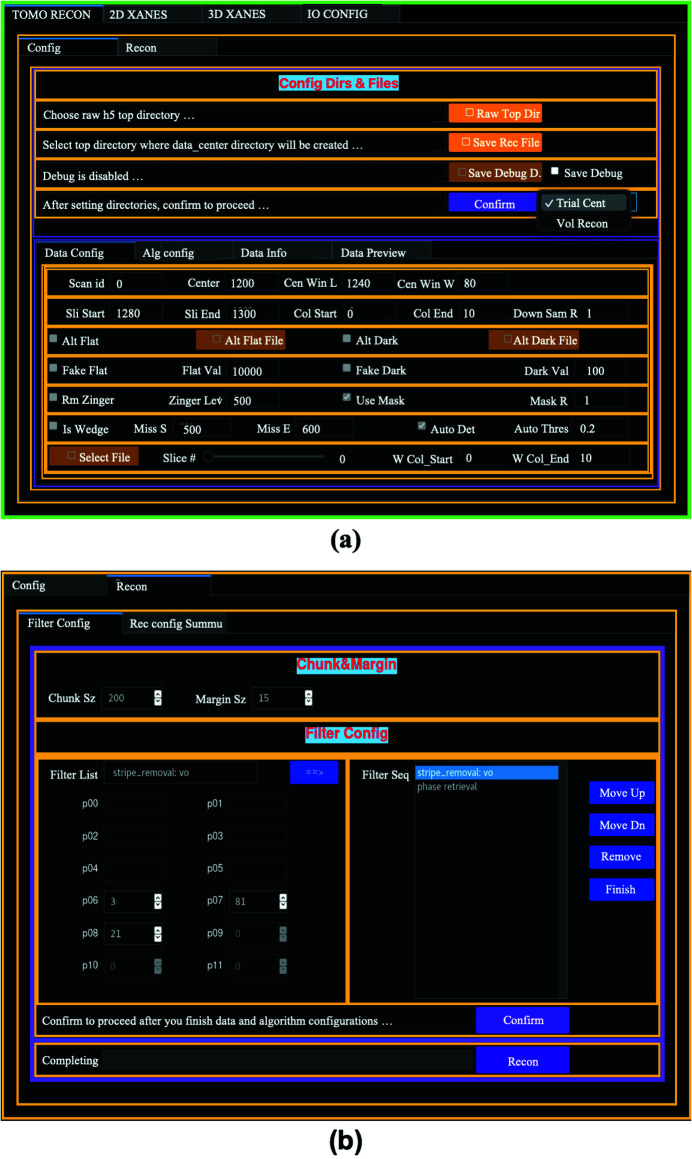
Tomography GUI layout. (*a*) Analysis type and data configuration, and (*b*) filter configuration.

Fig. 3 ▸ shows the tomography GUI layout. As shown in Fig. 3 ▸(*a*), there are two data processing configuration options: ‘Trial Center’ and ‘Vol Recon’. To perform a tomographic reconstruction, one needs to find the rotation axis position relative to the projection images’ center column. The option ‘Trial Center’ allows users to reconstruct a single slice with different rotation centers. With this option, users can also quickly check the effects of different pre-processing operations and reconstruction algorithms on the data. The configuration starts with specifying the paths to the raw data file directory and the directory to save the trial reconstruction. After the directories are defined, the parameter configuration and tomography reconstruction tabs are enabled. Under ‘Config/Data Config’, the scan numbers of the available data files with the given file name patterns in the specified raw data directory are shown in a dropdown list. The user-defined data information items displayed under the ‘Config/Data Info’ tab refresh after a new scan number is selected. The projection images of the selected scan can be viewed in an *ImageJ* viewer with the widgets under the ‘Config/Data Preview’ tab. Users can perform the reconstruction to a region of interest (ROI) by defining the slice and column ranges under the ‘Config/Data Config’ tab. The trail reconstruction images of a user-defined slice are reconstructed at different rotation center positions with 0.5 pixel step size over a range that is defined by the starting position and the width of the range.

The tomography reconstruction in *TXM-Sandbox* is based on *TomoPy* (Gürsoy *et al.*, 2014 ▸). There are various tomography reconstruction algorithms available in *TomoPy*. Users can choose one of a few algorithms, including gridrec, SIRT, TV, and MLEM, and define the algorithm parameters under the ‘Config/Alg Config’ tab. There are almost always some types of imperfections in TXM tomography data, *e.g.* bad pixels, zingers and noise. Pre-processing the data can reduce the artifacts induced by the imperfections in the data. *TXM-Sandbox* groups a few pre-processing filters based on both *TomoPy* and *scikit-image* (van der Walt *et al.*, 2014 ▸) packages, *e.g.* phase retrieval, stripe removal, and denoising, under the ‘Recon/Filter Config’ tab. As shown in Fig. 3 ▸(*b*), users select a function from the filter list and set the function’s parameters in the left panel, then add it to the pre-processing queue in the right panel. The order of the different operations may affect the reconstruction results, so it is necessary to set a suitable sequence to apply the filters to the data. For example, stripe removal filters, in general, should be applied before phase retrieval or denoising operations. Users can easily alter the filters’ order in the queue with the ‘Move Up’ and ‘Move Down’ buttons.

*TXM-Sandbox* also provides tools to handle certain scenarios in which incomplete data are presented. In some cases, there are no corresponding flat-field and dark-field images acquired together with the samples’ projection images, which may occur in *in situ* measurements due to the form of the sample. Under ‘Config/Data Config’, users can specify alternative flat-field and dark-field images from either separate flat-field and dark-field measurements or create artificial flat-field and dark-field images with constant values. In another scenario, a sample might be too thick along certain directions, so the projection images along these directions have very low count levels. It renders the images at these angles unusable, so it causes missing angles in the tomography scans. Under ‘Config/Data Config’, users can either manually specify the range of the missing angles or automatically determine the missing angles by discriminating the row-averaged gray values according to a threshold. However, tomography reconstructions with missing angles will suffer from missing-angle artifacts that change the gray values in the reconstructed slice images. In 3D XANES analysis, the local spectra depend on the local gray values at different X-ray energies. It has been shown that the difference between the altered gray values due to the missing angles and the gray values calculated without missing-angle artifacts is roughly a constant depending only on the sample geometry and the missing angle range (Xiao *et al.*, 2010 ▸). This suggests the XANES spectrum obtained from the tomography scans with missing angles of a sample at different X-ray energies only differs from that obtained from an ideal 3D XANES measurement by a vertical offset, provided the missing angle ranges in all the tomography reconstructions at different X-ray energies are the same. In *TXM-Sandbox*, users can specify one tomography dataset from the 3D XANES measurement for calculating the missing angle range. The same missing angle range is excluded from all the reconstructions at different X-ray energies.

Once all the configurations are done, users can check the summary of the configuration, make necessary changes, and confirm to proceed to the reconstruction step. After the trial reconstruction finishes, the image stack from the reconstruction will be displayed in an *ImageJ* viewer. Users will need to inspect the images to find the image with the least off-center artifacts (Gürsoy *et al.*, 2014 ▸) and confirm the best image under ‘Config/Data Preview’. The corresponding rotation center and reconstruction parameters will then be logged in a JSON file in the raw data file directory. Multiple raw data files can be processed in sequence under the ‘Trial Center’ option. The rotation center and reconstruction parameters for each raw data set are saved in the same JSON file, which can then be used as a configuration file for the volume reconstructions of these raw data files in batch with the ‘Vol Recon’ option in Fig. 3 ▸(*a*).

Under ‘Vol Recon’, users need to specify the configuration file (created during the ‘Trial Center’ step) for the volume reconstructions. *TXM-Sandbox* takes most configuration items from this file except for the slice range (which is usually limited to a very small range in trial step). Users can change this range for the batch volume reconstructions. Users can also configure the chunk size according to the available memory in the data processing computer. The chunk size determines how many image slices can be reconstructed at a time. For a computer with smaller memory, a smaller chunk size will reduce the memory footprint during the calculation. For the filtering operations on the projection images that involve manipulations in Fourier space, the top and bottom slices in a chunk can be affected by artifacts due to the boundary effects from the operations in Fourier space. Such boundary effects will cause inconsistent gray value levels in the affected slice image. To remove the boundary effects, a certain number of top and bottom slices in a chunk are thrown away. Users can specify a marginal size to determine how many slices should be thrown away. The default value for this marginal size is 15, which is is good for most pre-processing operations. The batch reconstructions will go through all the datasets defined in the configuration file. The reconstructed tomography data will be saved as a series of slice image files in TIFF format in a folder for each raw tomography dataset. The names for the reconstruction folder and files bear the raw data file name with a prefix ‘recon_’, so the raw and reconstructed data are associated with the raw data file names. Compared with saving the reconstruction of one dataset into a single file, saving the reconstruction into a series of slice image files provides users with better flexibility to view the results. A tomography reconstruction dataset is usually tens of gigabytes in size, which is usually too large to be read into a computer’s memory. On the other hand, a series of slice images can be easily viewed as a virtual stack in *Fiji/ImageJ* that requires limited computer memory usage.

### 3.3. 2D/3D XANES GUI layout

**Figure 4:**
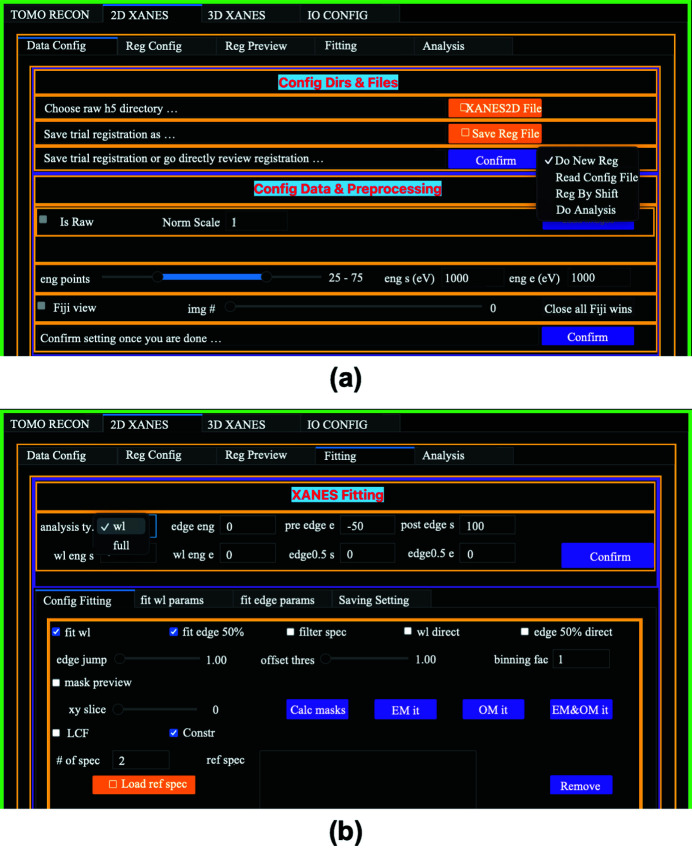
2D XANES GUI layout. (*a*) Analysis type and data configuration, and (*b*) XANES fitting configuration.

The TXM-XANES data are acquired by taking 2D radiographic images (2D XANES) or 3D tomography scans (3D XANES) of a sample at different X-ray energies across the absorption edge of a concerned element in the sample (Spence *et al.*, 2021 ▸). Once the 2D images/3D volumes are aligned, one spectrum at each pixel/voxel is obtained. The analysis of the spectra provides the oxidation state distribution of the concerned element inside the sample. Fig. 4 ▸ shows the 2D XANES GUI layout. As shown in Fig. 4 ▸(*a*), the operations are organized in a series of tabs: ‘Data Config’, ‘Reg Config’, ‘Reg Review’, ‘Fitting’, and ‘Analysis’. Like the tomography GUI layout, it starts with the data file directory configuration with which users specify where the raw data file is and where to save the analysis results. Under the ‘Data Config’ tab, users can preview the 2D XANES images in an *ImageJ* viewer and decide whether the images from partial or entire energy range will be used in the XANES analysis. In TXM-XANES experiments, due to the acquisition time, the sample position may drift within the detector field of view. It is necessary to register the images to guarantee the signals from the same location of the sample are assembled into a spectrum. Image registration is thus a critical step in TXM-XANES data processing. *TXM-Sandbox* integrates a few image registration methods, including the popular phase correlation method available in *scikit-image* and the Python wrapper to the TurboReg plugin in *Fiji* (https://pypi.org/project/pystackreg/), for aligning the sample images under the ‘Reg Config’ tab. A recently developed method (Xiao *et al.*, 2021 ▸), which is based on the minimization of the total variance (TV) of the difference map between two images with a multi-resolution computation approach, is highly efficient in such tasks. Compared with other alignment algorithms, the new method is insensitive to the varying backgrounds and contrasts that are typical in XANES images. Under the ‘Reg Config’ tab, users can also define an ROI before the registration. This not only helps to reduce the computation time but also separates the regions in the images that may have different relative drifts during the measurements. Once the configuration is done, users can run the trial registration. It is always a good practice to inspect the registration results before XANES fitting. The difference map between a pair of images can highlight the misalignment between the images, especially in the edge regions. Under the ‘Reg Review’ tab, users can go through image pairs and correct the mis-registered image manually by tweaking the relative shifts between the image pairs. It is demonstrated that the registration results with the new TV-based method rarely need manual adjustments in TXM-XANES cases. Once the shifts between image pairs based on the trial registration results and manual tweaking are confirmed, the entire set of XANES images will be translated to generate the aligned image stack for XANES spectrum fitting analysis.

TXM-XANES analysis differs from the conventional XANES analysis in three aspects. Firstly, the pixel-wise TXM-XANES data quality is usually not as good as that in the conventional XANES spectra. Compared with the conventional XANES spectra in which the absorption signal comes out from a sample area typically of ∼10^4^ to 10^6^ µm^2^ scale, the TXM-XANES signal at an individual pixel is from areas of ∼10^−3^ µm^2^ scale. Secondly, the acquisition time at each energy point in TXM-XANES cases is longer than that in the bulk XANES cases to obtain reasonable statistics on the signals from small sample areas. Thirdly, there are typically 10^6^ to 10^9^ spectra from each TXM-XANES measurement. Thus, automatic and reliable data analysis methods are required in TXM-XANES.

Considering these differences, many conventional XANES analyses cannot be applied in TXM-XANES. For instance, the pre-edge fitting in the conventional XANES is based on a trial-and-error approach that is not a feasible option for processing a large number of high-noise spectra in TXM-XANES. In an XANES spectrum, the signal (absorption coefficient) is high around the white-line position, but low in the pre-edge region; thus, the spectrum in the pre-edge region tends to have a lower signal-to-noise ratio. Therefore, one option for fast batch analysis is to focus on the white-line position shifts. Although the white-line position of a XANES spectrum is not always linearly correlated with the concerned element’s valence state, in a typical TXM-XANES experiment XANES spectra of the element’s different valence states in known samples are usually measured and can be used as references. Therefore, the white-line position of a sample can be used as a feature of its XANES spectrum for tracking its valence state change. In the cases of cathode materials in lithium ion batteries, most interested elements, including Ni and Co in LiNi_*x*_Mn_*y*_Co_1–*x*–*y*_O_2_ (NMC) materials and Co in LiCoO_2_, are such cases. It has been shown that tracking the white-line position provides useful information on the heterogeneous valance state distributions at the electrode particle level (Henderson *et al.*, 2014 ▸; Wolf *et al.*, 2017 ▸; Xue *et al.*, 2021 ▸). This approach does not require pre-edge background subtraction and post-edge background normalization. Thus, a TXM-XANES measurement can be made at a smaller set of energy points, typically less than 20 points, compared with that required in the full spectrum analysis. This method facilitates fast 2D/3D XANES measurements that are beneficial to *in situ* experiments. Alternatively, another option is to determine the edge energy as the energy where the normalized absorption χ equal 50% of the maximum (de Groot *et al.*, 2004 ▸), referred to as the edge-50 method hereafter. This approach requires the measurements over an energy range, including both the pre-edge background and post-edge background, to calculate the normalized absorption spectrum μ(*E*). Since the signals in the pre-edge range are poor, μ(*E*) may be offset by the error from the pre-edge background subtraction. Therefore, this method is more sensitive to the noise in the data than the white-line method and may give incorrect results if the data quality is not high. Nonetheless, the edge-50 method provides a physically more meaningful approach when the data quality is satisfactory. We also highlight that it is a good strategy to refer to the bulk XANES spectra for the element of interest to determine whether the edge-50 or the white line is more sensitive to its oxidation state.

Linear combination fitting (LCF) is a standard XANES analysis technique (Newville, 2014 ▸). In the conventional XANES cases, LCF gives precise valence state information with appropriate reference spectra. In TXM-XANES, LCF may generate results with significant errors if the data noise is high. LCF is implemented as one analysis option in *TXM-Sandbox*. However, users need to pay attention to the LCF results due to the above-mentioned signal-to-noise issue.

In fitting TXM-XANES data, as shown in Fig. 4 ▸(*b*), users can choose between two options: ‘wl’ for the data only available at the energy points around the white-line position, and ‘full’ for the data available at the energy points over the full XANES regime. With the ‘wl’ option, only white-line peak and rising edge fittings are available for TXM-XANES data analysis. With the ‘full’ option, all types of TXM-XANES analyses, including the white-line peak, edge position determined by derivative, edge-50, and LCF, are permitted. Users can choose all or part of the available analysis types. The items available for saving under the ‘saving setting’ update are accordingly based on the selected analysis type. There are also two dedicated tabs designed for the white-line peak and rising edge fitting configurations. Users can specify the fitting functions from a predefined function pool and define the parameters’ initial values.

There are other analysis tools available in *TXM-Sandbox*. Two XANES masks based on the spectrum sanity check at each pixel are proposed by Liu *et al.* (2012 ▸). It is demonstrated that these masks are useful to discriminate the pixels that have good signals from those with bad signals. In *TXM-Sandbox*, users can interactively adjust the mask parameters by previewing the sample images with the masks.

The 3D XANES GUI layout is mostly the same as the 2D XANES GUI. The main difference is in the layout under the ‘Data Config’ tab that is to accommodate the different data organizations for 2D and 3D XANES data. In the 2D XANES case, the sample images at different energies and the energy point array are in the same HDF5 file. Whilst in the 3D XANES case, the sample images, which are the tomography reconstruction images at different energy points, are saved in a series of files in different folders. For XANES analysis, it needs to access the sample images in the tomography reconstruction folders and the associate energy values in the raw data files. Therefore, users need to specify both the directories to both the raw data files and reconstruction folders.

## 4. Application examples

A few application cases on tomographic, 2D XANES and 3D XANES data processing are demonstrated in this section. All the data used here were taken at the Full-Field X-ray Imaging beamline of National Synchrotron Light Source II at Brook­haven National Laboratory. The data were saved in the default HDF5 format.

### 4.1. Tomography reconstructions with and without missing angle removal

**Figure 5:**
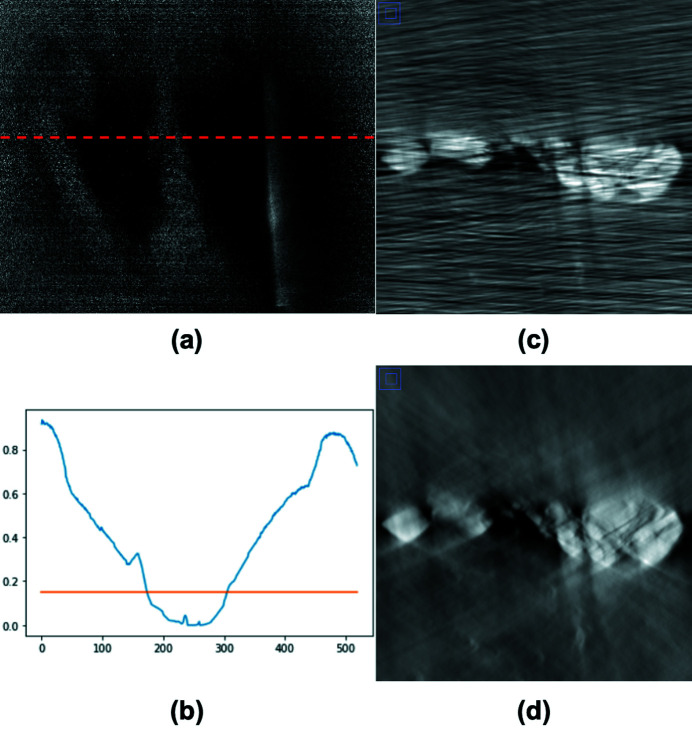
(*a*) The projection image of a battery electrode sample at an angle when the electrode is parallel to the X-ray beam. (*b*) The mean normalized value averaged along the row direction as a function of the projection angle index. The mean value drops close to zero in an angle range. (*c*) A reconstructed slice image with all the projection images over 180° angle range. The stripe lines are due to the low signals in the angle range. (*d*) The reconstructed slice image with the projection images except that in the angle range in which the mean values are lower than the threshold marked in (*b*).

Fig. 5 ▸ illustrates the missing angle effects and the missing-angle determination processing. The X-ray path through a battery electrode sample becomes very long when its laminar plane is roughly parallel to the X-ray direction. A projection image within this angle range has a very low signal level, as seen in Fig. 5 ▸(*a*). Fig. 5 ▸(*b*) plots the normalized row-averaged gray value along a line as a function of the rotation angle. It shows that the projection images in an angle range around 90° have an average transmission of less than 15%, which corresponds to a signal-to-noise ratio of *xy* in the current case. Without excluding the projection images with low signal levels, the reconstructions with the Gridrec algorithm (Dowd *et al.*, 1999 ▸) show strong streaking artifacts, as shown in Fig. 5 ▸(*c*). In comparison, Fig. 5 ▸(*d*) is the reconstruction excluding the projection images in the shaded angle range in which the images have row-averaged attenuation lower than 15%. The reconstruction in Fig. 5 ▸(*d*) presents no streaking artifacts but moderate missing-angle artifacts that change absolute gray values locally across the sample. The missing angle artifacts can be further reduced by using algebraic or machine-learning-based reconstruction techniques.

### 4.2. Comparison between two XANES analysis methods in 2D cases

**Figure 6:**
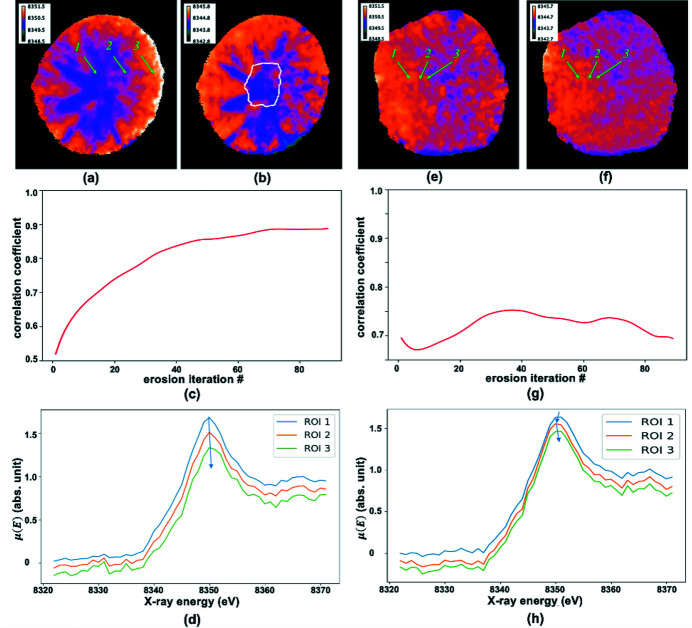
Comparison between analysis results with white-line and edge-50 methods. (*a*) White-line and (*b*) edge-50 maps of a NMC811 particle; the correlation between (*a*) and (*b*) as a function of ROI with the size shrinking from the outline of the particle to that marked in (*b*) is shown in (*c*). (*d*) Spectra of three ROIs indicated in (*a*). The white-line peak position shifts toward higher energy when the ROI moves from the inner region to the outer region, and the corresponding edge-50 positions show the same trend. (*e*)–(*h*) Results of a second NMC811 particle. Although the white-line position of ROI 2 is lower than ROIs 1 and 3, its edge-50 position is highest in three ROIs (ROI 1: 8344.8 eV; ROI 2: 8344.8 eV; ROI 3: 8345.7 eV).

Since it is impossible to manually inspect the large number of spectra in TXM-XANES, the reliability of the analysis relies on the robustness of the analysis methods. Fig. 6 ▸ presents the analysis results of 2D XANES at the Ni edge of two NMC811 particles. In the first example shown in Figs. 6 ▸(*a*)–6(*d*), panels (*a*) and (*b*) are the white-line and edge-50 position maps of an NMC811 particle that represent Ni’s inhomogeneous valence state distributions across the particle. The white-line peak fitting is based on a second-order polynomial function, and the rising edge fitting is based on a third-order polynomial function. The correlation between two maps as a function of the ROI size is shown in Fig. 6 ▸(*c*). The ROI used in the calculation is generated by shrinking the region by one pixel in each step, starting from the particle outline border until the final region marked in Fig. 6 ▸(*b*). It is found that the white-line and edge-50 maps are positively correlated but also show some differences. In a 2D projection image through a ball-shape sample, the sample’s attenuation to X-ray increases from the outline border to the center, so the XANES signal becomes greater from the outer region to the inner region of the particle. Therefore, shrinking the ROI in the correlation calculation removes the noisier data in the outer region and improves the correlation between the white-line and edge-50 maps. Fig. 6 ▸(*d*) displays the spectra in the three selected ROIs marked in Fig. 6 ▸(*a*). The noise level in the energy range before the pre-edge clearly increases from the center toward the border (ROI 1 → ROI 3). Nonetheless, the white-line peak positions (ROI1: 8350.0 eV; ROI2: 8350.3 eV; ROI3: 8350.7 eV) shows a clear trend shifting toward higher energy from the center region to the border region. In comparison, edge-50 positions (ROI1: 8344.4 eV; ROI2: 8344.5 eV; ROI3: 8345.0 eV) also show a similar trend.

The same comparisons for a second particle are presented in Figs. 6 ▸(*e*)–6(*h*). The correlation between the white-line map Fig. 6 ▸(*e*) and edge-50 map Fig. 6 ▸(*f*) is lower than that in the first example, as shown in Fig. 6 ▸(*g*). In particular, the local details in Figs. 6 ▸(*e*) and 6 ▸(*f*) are different. Fig. 6 ▸(*h*) plots the spectra in the three regions of interest marked in both Figs. 6 ▸(*e*) and 6 ▸(*f*). Whilst the white-line position of ROI 2 (8350.5 eV) is lower than that of ROI 1 (8350.7 eV) and ROI 3 (8350.6 eV), the edge-50 position of ROI 2 (8344.8 eV) is higher than other two regions (ROI 1: 8344.8 eV; ROI 3: 8345.7 eV). Thus, the correlation between the two maps fluctuates when the ROI shrinks from the outline border region toward the center region. This example suggests that the white-line method is more sensitive than the edge-50 method in terms of revealing the difference in the noisy TXM-XANES spectra for the Ni *K*-edge. It is important to note that the type of element also influences whether white-line or edge-50 is more appropriate to reveal oxidation states. White-line or edge-50 positions of an XANES spectrum by themselves are not enough to provide the valence states of an element in the interested material. It is necessary to combine it with the bulk XANES characterizations in which the signature differences in white-line or edge-50 maps at different states can be linked to the specific valence states characterized by the bulk XANES.

### 4.3. 3D XANES white-line fitting showing non-uniform valence distribution

**Figure 7:**
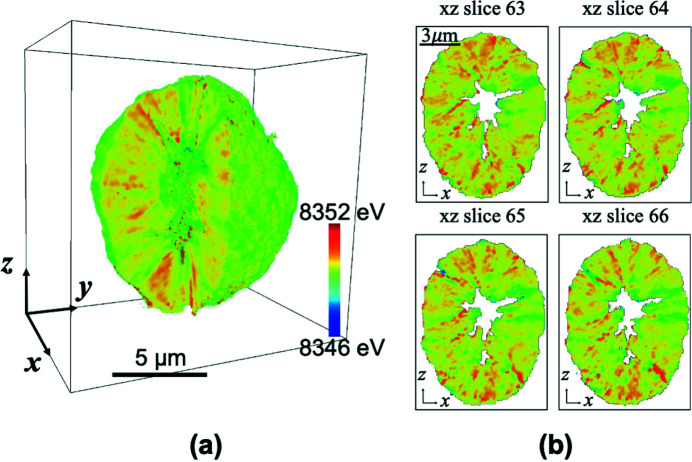
White-line peak position map of a NMC811 particle. (*a*) 3D rendering of the white-line peak position distribution. (*b*) Four slices parallel to the *xz* plane show slow variation in the *y* direction

TXM-XANES can be extended to 3D by conducting tomography scans at a set of energy points across a concerned element’s absorption edge. Fig. 7 ▸ presents an example of the white-line position 3D distribution in an NMC811 particle in the charged state. This particle is about 10 µm in diameter, with a void in the center. For this secondary particle, the rod-shaped single-crystal primary grains are radially aligned. The (003) planes are along the long axes of the rod grains that provide fast Li-ion diffusion channels for Li-ion insertion/extraction (Xu, Jiang *et al.*, 2020 ▸). During the charging process, Li-ions could be transported quickly between these planes from inside to outside of the particle whilst the diffusions between different grains are minor. As shown in Fig. 7 ▸(*a*), some grains developed a higher level of valence states than others. The non-uniform deli­thia­tion across the particle could induce intra-grain stress. If the stress could not relax but gradually accumulate in the following repeating cycling processes, the particle might generate microcracks that can deteriorate the material’s performance. With the correlative morphological-chemical information provided by 3D XANES, we can gain insights on, for example, how the particle level structures correlate to the overall battery performance, which are critical to designing new batteries with elevated properties (Spence *et al.*, 2021 ▸).

## 5. Summary

A Python-based software *TXM-Sandbox* for tomography and full-field XANES data analysis is developed. The software provides a user-friendly graphic interface in *Jupyter Notebook* and rich image visualization capabilities available in *Fiji/ImageJ*. The integrated *Jupyter Notebook* environment allows users to easily access the intermediate data in the analysis that facilitates the flexibility of expanding the data analysis beyond the built-in functions provided by *TXM-Sandbox*.

*TXM-Sandbox* currently only includes tomography and image-based XANES processing and certain pre-processing functions. For post-processing based on the outputs from *TXM-Sandbox* results, users can choose other suitable packages and software. For instance, *scikit-image* and *OpenCV* provide rich image analysis tools, *scikit-learn* provides machine learning based analysis tools, and *Fiji/ImageJ* and *ORS Dragonfly* provide image visualization and segmentation functionalities. In the future, *TXM-Sandbox* can implement interfaces to the Python compatible packages based on users’ demands.

Along with the world-wide rapid synchrotron source developments, the data generation rate increases quickly. *TXM-Sandbox* still involved human intervention steps in tomographic and TXM XANES data analyses. It is partially due to the complexity in TXM data in which the contrast in images changes dramatically as a function of X-ray energy, samples’ sizes are almost always larger than the TXM field of view, and missing-angle issues appear often in tomographic scans. More advanced data processing and analysis methods including artificial intelligent and machine-learning-based methods are needed to handle large amounts of data with variant properties. *TXM-Sandbox* can be used as a framework for integrating further data analysis approaches.
